# Enhancing awareness and uptake of home-based care services during the coronavirus disease 2019 pandemic in Zambia

**DOI:** 10.4102/jphia.v16i4.1627

**Published:** 2025-12-17

**Authors:** Kelvin Mwangilwa, Cephas Sialubanje, Nyuma Mbewe, Naeem M.I. Dalal, Oliver Mweso, Stephen Longa Chanda, Musole Chipoya, Roureen P. Landson, Chilufya S.A. Mulenga, Moses Mwale, Moses Banda, Vivian M. Mwale, Priscilla N. Gardner, Geoffrey Mutiti, Lilian Lamba, Charles Chileshe, Peter Funsani, Davie Simwaba, Paul M. Zulu, Raymond Hamoonga, Malambo Mutila, Innocent Hamuganyu, Jonathan Mwanza, Olive Chiboola, Nyambe Sinyange, Muzala Kapin’a, Nkomba Kayeyi, Fred Kapaya, Mazyanga L. Mazaba, Roma Chilengi

**Affiliations:** 1 Zambia National Public Health Institute, Lusaka, Zambia; 2 School of Public Health, Levy Mwanawasa Medical University, Lusaka, Zambia; 3 World Health Organization, Lusaka, Zambia; 4 Southern Africa Institute for Collaborative Research and Innovative Organization (SAICRIO), Lusaka, Zambia; 5 Africa CDC Eastern Africa Regional Coordinating Centre, National Kenyata Hospital, Nairobi, Kenya

**Keywords:** home-based care, COVID-19, patient uptake, healthcare utilisation, socio-economic factors, awareness, Zambia

## Abstract

**Background:**

The COVID-19 pandemic placed pressure on health systems, exposing workforce shortages and prompting innovative strategies to manage patients with mild to moderate symptoms. Home-based care emerged as a practical approach to reduce facility burden while maintaining quality care.

**Aim:**

To assess the implementation and acceptability of the COVID-19 home management model in Zambia.

**Setting:**

The study was conducted in 11 purposively selected districts with high levels of home-based management.

**Methods:**

A comparative cross-sectional study was conducted. Data were collected in June 2023 and September 2023 from 566 individuals with confirmed COVID-19 eligible for home management, sampled systematically from health facility line lists. Descriptive statistics summarised participant characteristics, and multivariable logistic regression identified factors associated with accepting home-based care.

**Results:**

Sixty per cent participants were female, with a median age of 28 years. Awareness of the home management model (adjusted odds ratio [AOR] = 5.11; 95% confidence interval [CI]: 2.61–10.0), income between 600 and 1000 kwacha (AOR = 2.64; 95% CI: 1.10–6.85), and perceiving the model as effective (AOR = 7.88; 95% CI: 3.56–18.3) increased odds of acceptance, while formal employment reduced it (AOR = 0.38; 95% CI: 0.18–0.78).

**Conclusion:**

Home-based care is a strategy for easing health system pressure. Strengthening awareness and addressing socio-economic barriers could increase uptake in Zambia.

**Contribution:**

This study contributes new evidence on the determinants of home-based care uptake within a low-resource context. The study provides actionable insights for policymakers and programme implementers seeking to strengthen community-based models of care.

## Background

Global healthcare systems faced significant strain during the coronavirus disease 2019 (COVID-19) pandemic, putting acute pressure on frontline health workers and necessitating creative workforce strategies and patient management approaches.^1,2,3^ Households in developing countries were noted to have the highest vulnerabilities, often unable to access necessary care because of financial constraints and transportation challenges. To mitigate hospital costs while ensuring high-quality patient care, home-based treatment emerged as a viable initiative for managing mild to moderate COVID-19 cases, effectively reducing the burden on healthcare.^4,5^

In an effort to help countries relieve congestion in health facilities, the World Health Organization (WHO) launched the home-based Isolation and Care (HBIC) strategy.^6^ The strategy guides the appropriate monitoring of patients and timely detection of those with risk factors for disease severity and progression. It was aimed at supporting members of the public to avoid late presentation at healthcare facilities, thereby decreasing morbidity and mortality among COVID-19 patients. Appropriate implementation would also help break the chain of COVID-19 transmission.^7^

Zambia adopted a home-based care model for the management of asymptomatic and mildly symptomatic COVID-19 patients who were without underlying medical conditions or comorbidities from May 2020.^8,9^ On the other hand, the WHO made home-based care on recommendations for COVID-19 patients.7 The strategy relies on local resources to reduce pressure on the healthcare system. When well implemented, this strategy has the potential to increase the efficiency of management of COVID-19 patients as well as the healthcare system in general.^1,2^

In May 2020, Nakonde district in Zambia experienced a significant surge in COVID-19 cases, recording 400 infections within three days.^10^ Healthcare systems experienced significant strain during the COVID-19 pandemic, primarily because of the rapid influx of patients requiring medical attention. This sudden surge overwhelmed health facilities and placed immense pressure on frontline health workers, exposing critical gaps in health workforce capacity and system preparedness.^11,12^ In low- and middle-income countries, the situation was further exacerbated by limited financial resources and transportation challenges, which hindered access to care for many households. To address these challenges, health authorities adopted innovative approaches to workforce deployment and patient management, including home-based care strategies to alleviate the burden on health facilities. This initiative allowed patients with mild symptoms to receive care at home, thereby reserving hospital resources for more severe cases.^13^ The approach was later incorporated into the national COVID-19 response plan and expanded to other parts of the country.^4^

Evidence from hospital-at-home models for COVID-19 patients, including telemedicine consultations and remote monitoring, has shown that these programmes can deliver outcomes comparable to, or better than, conventional hospital care.^4,5,6,7,14^ However, the ability of patients to use home-based care services may be impacted by disparities in access to technology, education and income. Socio-economically disadvantaged groups are more susceptible to infections and more likely to experience severe illness progression, which can hinder their ability to use home-based care.^15^ Furthermore, disparities in technology, like telemedicine, may affect access to home-based care.^16^

The success of the home-based care strategy requires the usual resources, including medical equipment and financial resources, which are usually unavailable. If effectively implemented, the strategy has the potential to increase the efficiency of the management of COVID-19 patients as well as the healthcare system in general for other diseases.^7,9^ Understanding the factors influencing uptake of home-based care is crucial for optimising its implementation. This study aimed to identify factors influencing the uptake of home-based care services, to make policy recommendations for similar models in future infectious disease outbreaks in resource-limited settings like Zambia.

## Research methods and design

### Study design

This study used a cross-sectional design to assess the implementation of the COVID-19 home management strategy in Zambia and determine the factors influencing uptake of the model.

### Study setting

The study was conducted in 11 districts purposively selected from the 10 provinces of the country. Zambia is administratively divided into 10 provinces, followed by 116 districts and health facilities. The 11 districts were purposively selected because they reported the highest numbers of confirmed COVID-19 cases during the study period. To enhance representativeness, selection also considered geographic distribution and population diversity to ensure that all 10 provinces of the country were included. However, at the patient level, a systematic random sampling process was applied using the line list provided by each District Health Office (DHO) to select study participants within the chosen districts.^17^ This approach enhanced the representativeness and reduced potential selection bias at the individual participant level.

### Study participants

**FIGURE 1:**
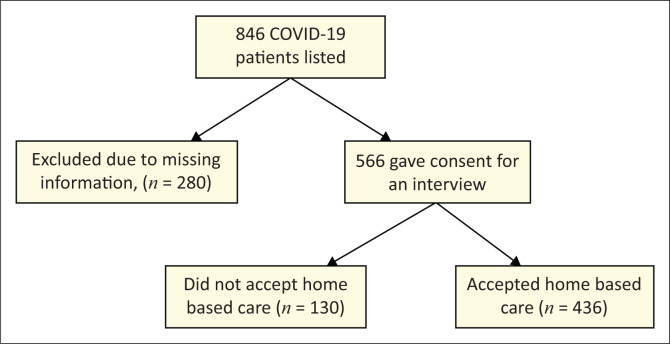
Flow chart showing the sampling process and participant inclusion. Out of 846 listed coronavirus disease 2019 patients, 566 provided consent for interview, of whom 436 accepted home-based care and 130 did not.

Study participants comprised COVID-19 patients who were managed at home between June 2023 and September 2023 (Figure 1).

### Sampling design and sampling process

The districts were purposively selected because they reported a relatively high burden of COVID-19 cases compared to other districts among the 116 districts of Zambia. In this study, a high number of cases was defined using epidemiological thresholds recommended by the WHO. According to WHO, high-transmission areas are typically defined as those with a test positivity rate greater than 5% or incidence greater than 100 cases per 100 000 population per week.^18,19,20^ Similarly, studies in Zambia and the region have used district-level incidence thresholds to classify high-burden areas for surveillance and intervention prioritisation.^8^ The districts represented were from the 10 provinces of Zambia.

### Sample size determination

A sample size calculation was conducted to determine the number of participants required to compare the proportion of individuals who accepted versus those who did not accept COVID-19 home-based care management. These two groups (Group 1: accepted home-based care; Group 2: did not accept home-based care) were defined based on patients’ reported decisions regarding the home isolation programme.^21^ Based on findings from prior studies and pilot data, it was assumed that approximately 60% of patients advised to isolate at home would accept home-based care, while 40% would not. Using a two-sided Z-test for two proportions, with a significance level (*α*) of 0.05 and statistical power of 80%, the minimum required sample size was estimated at 98 participants per group (total *n* = 196) to detect a significant difference between the two proportions (see Equation 1). To account for potential non-response or incomplete data, a 10% buffer was added, resulting in a final target sample size of 108 per group (*n* = 216 in total). In the absence of prior studies quantifying uptake of home-based care among COVID-19 patients, the sample size was estimated using conventional assumptions for comparing two proportions. A moderate difference of 20% points between groups was assumed (*p*_1_ = 0.60 and *p*_2_ = 0.40). This approach is commonly recommended when empirical data are limited, as it provides a conservative and realistic estimate of the required sample size.^22,23,24^


$$\text{n}=\frac{{\left[{\text{Z}}_{1-\alpha /2}\sqrt{2}{\text{P}}^{-}{\text{Q}}^{-}-{\text{Z}}_{1-\beta }\sqrt{{\text{P}}_{1}}Q_{1}+{\text{P}}_{2}\;{\text{Q}}_{2}\right]}^{2}}{{\left({\text{P}}_{2}-{\text{P}}_{1}\right)}^{2}}$$ [Eqn 1]

### Sampling process

Systematic random sampling was employed to select participants from the list of all COVID-19 patients who were managed under the home-based programme. The sampling frame comprised all patients recorded on the line list across the selected districts during the study period. The total number of eligible patients was first established, after which the sampling interval (k) was calculated by dividing the total number of patients (*N*) by the desired sample size (*n*).^25^ A random starting point between 1 and 3 was generated using a computer-based random number generator, and every kth patient in the ordered list was subsequently selected until the required sample size was reached. The database was exported into Microsoft Excel^®^, where patient identification numbers were arranged in ascending order according to the date of enrolment. The use of a computer-assisted approach ensured objectivity and minimised selection bias. In cases where selected records were incomplete or ineligible, the next consecutive record was included to maintain the sampling sequence and achieve the targeted sample size.

The health facility line list from the DHO was used as the sampling frame. Participants were drawn at random from the sampling frame in a systematic manner using a random interval for each district, given the number of eligible participants on the line list. The sampling interval was calculated by dividing the population size by the desired sample size. To ensure a self-weighing sample, a proportion of participants were selected from each district. That is, the number of selected participants varied according to the sampling frame in each area.

### Inclusion and exclusion criteria

To be included in the sample:

- Patients should have tested positive for COVID-19 by polymerase chain reaction (PCR) whether the patient had mild or no symptoms.
- Patients isolated in hotels and non-home settings were excluded from the study.

### Variables

The outcome variable uptake (uptake or not) was whether the participant received home-based care or not. Independent variables of interest were age, sex, educational level, employment status, head of home-based care management, whether the patient received encouragement to take home-based care or not, contact with healthcare worker or not, nearest health facility or not and monthly income.

### Data collection

A group of dedicated data collectors conducted interviews in 11 districts. The districts were purposively sampled because they had a high number of positive COVID-19 cases among 116 districts of Zambia. The field team comprised data collectors who were deployed in each district for data collection. These data collectors are fluent in the local languages and understand the culture of the communities. They were trained in research ethics,^26^ data collection questionnaire implementation and worked under the supervision of a team leader. Data collectors and supervisors were trained on how to complete the questionnaire on the tablet. Data quality was also controlled by close supervision, data cleaning and editing, and cross-checking of the completeness of the questionnaires. The questionnaire was pre-tested in similar settings, which were not part of the study area, and the necessary modifications were made to some items of the questionnaire.

### Data analysis

Data from 846 participants were cleaned in Microsoft Excel^®^.

Descriptive statistics were employed to summarise participant characteristics. Univariable and multivariable logistic regression were employed to determine the factors that predicted acceptance of home-based care management of patients. An investigator-led multivariable logistic regression was used to select variables that best predicted the outcome, accounting for the weighting. The variables for the final multiple regression model were chosen by first running the multiple logistic regression command with all of the predictor variables, then removing one by one the predictor variables with the highest *p*-values from the model until only those predictor variables that best predicted the outcome remained in the model. Finally, based on Akaike’s Information Criterion and Bayesian Information Criterion (AIC and BIC) for the competing models, the best-fit model was chosen. The model that had the lowest AIC and BIC values in comparison to other models was picked. The 95% confidence intervals (CI) for the crude odds ratio (cOR) and adjusted odds ratios (aOR) were generated. A *p*-value less than 0.05 was regarded as significant. R statistical software (version 4.3.2; RStudio, Inc.) was used for all analyses.

### Ethical considerations

Ethical clearance to conduct this study was obtained from the University of Zambia Biomedical Research Ethics Committee (UNZABREC: REF. No. 4452 2023) and the National Health Research Authority (No. NHRA0004/19/10/2023). Anonymity and confidentiality were ensured because the data sets were de-identified; they contained identification numbers rather than participants’ names. All information and survey findings were kept private and securely maintained. Paper records with household identifiers, household and locator information were stored apart from interview records in locked offices and a secure cabinet. The Zambia National Public Health Institute (ZNPHI) was in charge of ensuring that all documents were securely stored.

## Results

### Demographic characteristics of the participants

A total of 566 participants were included in our analysis. Most participants (60%) were female and aged between 35 years and 49 years; 170 (33%), with a median age of 28 years (IQR: 28–50). Nearly half (48%) of the participants had attained secondary education. Additionally, almost two-fifths (43%) of the participants were unemployed.

The majority (88%) of the respondents knew about home management of COVID-19 patients. Of these, 436 (83.7% had received home-based care; *n* = 85 [16%]) did not receive any home-based care. On monthly income during home-based care, 158 (30%) of the participants earned above 3000.00 Zambian Kwacha (approximately $100.00). The majority (75%) of the participants had sought care from the healthcare worker upon experiencing symptoms of COVID-19. Most (90%) of the participants lived less than 10 km from a health facility (Table 1).

**TABLE 1:** Bivariate analysis of background and social demographic characteristics of home-based care patients.

Characteristic	Overall (*N* = 566)		Received home-based care				*p*-value*
			No (*n* = 130)		Yes (*n* = 436)		
	*n*	%	*n*	%	*n*	%	
**Age group (years)**	-	-	-	-	-	-	0.179
≤ 14	13	2	11	1	12	3	-
15–24	72	13	9	11	58	13	-
25–34	144	26	39	34	105	24	-
35–49	190	33	40	35	140	32	-
50+	147	26	31	19	121	28	-
**Sex**	-	-	-	-	-	-	0.252
Female	334	60	56	54	278	61	-
Male	232	40	49	46	183	39	-
**Educational level**	-	-	-	-	-	-	0.177
Primary	73	13	16	7	62	14	-
Secondary	272	48	52	49	220	48	-
Tertiary	221	39	47	44	169	38	-
**Employment status**	-	-	-	-	-	-	0.032
Unemployed	240	43	37	32	198	45	-
Self-employed	147	26	33	27	124	26	-
Employed	169	31	45	41	129	28	-
**Heard of home-based care**	-	-	-	-	-	-	< 0.001
No	80	12	39	34	46	8	-
Yes	476	88	76	66	410	92	-
**Encouraged for home-based care**	-	-	-	-	-	-	0.999
No	109	16	34	16	75	16	-
Yes	457	84	81	84	366	84	-
**Monthly income in Kwacha**	-	-	-	-	-	-	0.168
< 500.00	161	29	37	32	124	28	-
600.00 to 1000.00	106 (18)	18	20	12	86	20	-
1500.00 to 3000.00	136	22	26	19	100	23	-
Above 3000.00	163	30	47	38	126	29	-
**Contact with health care worker**	-	-	-	-	-	-	0.161
No	151	25	47	32	104	24	-
Yes	415	75	83	68	332	76	-
**Nearest health facility**	-	-	-	-	-	-	0.943
Above 10 km	71	10	29	11	42	10	-
0 km – 10 km	495	90	101	89	394	90	-

* , Pearson’s Chi-squared tests.

There was a significant difference in acceptance of home-based care management services among patients who were employed, unemployed or self-employed (*p* = 0.032). Similarly, there was a significant difference (*p* < 0.001) in the acceptance of home-based care management between patients who had heard about the model and those who had not. Nonetheless, there was no significant difference in acceptance of home-based care management among participants of a particular age group, whether male or female, difference in educational status, monthly income, whether the participants were encouraged to use home-based care or not, whether they went to the health facility or not and whether the participant lived less than 10 km or not from the health facility evidenced by *p*-value greater than 0.05 (Table 1).

### Predictors of home-based management of coronavirus disease 2019 patients

Table 2 presents the final multivariable logistic regression model comparing COVID-19 patients uptake of home-based care management with those who did not. The model demonstrated a good fit to the data. Employed participants had significantly lower odds of uptake of home-based care management compared to unemployed participants, by approximately 62% (AOR = 0.38; 95% CI: 0.18–0.78). Conversely, participants who had previously heard about home management of COVID-19 patients were 6.5 times more likely to uptake home-based care compared to those who had never heard about it (AOR = 6.50; 95% CI: 3.56–11.9). Similarly, those with a monthly income between 600.00 and 1000.00 Zambian Kwacha had 2.6 times higher odds of uptake of home-based care compared to those earning less than 600.00 Kwacha per month (AOR = 2.64; 95% CI: 1.10–6.85). In addition, participants who perceived that the home management model worked well had 4.9 times higher odds of being managed at home compared to those who did not (AOR = 4.90; 95% CI: 2.74–8.68).

**TABLE 2:** Predictors of home-based management of coronavirus disease 2019 patients.

Characteristic	Unadjusted			Adjusted		
	OR	95% CI	*p*-value	AOR	95% CI	*p*-value
**Sex**						
No	-	-	-	-	-	-
Yes	0.74	0.46, 1.18	0.206	0.64	0.37, 1.10	0.107
**Educational level**						
Primary	-	-	-	-	-	-
Secondary	0.48	0.18, 1.11	0.114	0.43	0.15, 1.09	0.097
Tertiary	0.43	0.16, 1.00	0.068	0.69	0.21, 2.00	0.506
**Employment status**						
Unemployed	-	-	-	-	-	-
Self-employed	0.68	0.37, 1.24	0.202	0.80	0.40, 1.60	0.517
Employed	0.48	0.28, 0.83	0.010	0.38	0.18, 0.81	0.014
**Heard of home-based care**						
No	-		-	-		-
Yes	5.75	3.27, 10.1	< 0.001	5.11	2.61, 10.0	< 0.001
**Encouraged for home-based care**						
No	-	-	-	-	-	-
Yes	1.03	0.53, 1.88	0.924	1.10	1.05, 1.15	0.022
**Monthly income in Kwacha**						
< 500.00	-		-	-	-	-
600.00–1000.00	1.87	0.89, 4.25	0.113	2.64	1.10, 6.85	0.036
1500.00–3000.00	1.36	0.70, 2.71	0.369	1.84	0.83, 4.19	0.139
Above 3000.00	0.86	0.48, 1.51	0.596	1.15	0.52, 2.52	0.734
**Cared for a patient at home**						
No	-	-	-	-	-	-
Yes	0.61	0.38, 1.00	0.047	0.69	0.39, 1.25	0.217
**Model worked well**						
No	-	-	-	-	-	-
Yes	4.9	2.74, 8.68	< 0.001	7.88	3.56, 18.3	< 0.001

OR, odds ratio; CI, confidence interval; AOR, adjusted odds ratio.

Although participants’ education level and income were not statistically significant in the final model, they were retained based on prior empirical evidence showing that these factors are important predictors of acceptance of home-based care. Overall, the comparative analysis highlights that socio-economic factors and prior awareness significantly differentiated patients who accepted home-based care management from those who did not.

## Discussion

This study assessed factors influencing uptake of home-based care services for COVID-19 patients in Zambia. Our findings revealed that employment status, knowledge about home-based care management, monthly income and believing in the effectiveness of home-based care were predictors of acceptance of home-based care management.

The finding that employed participants were less likely to uptake home-based care aligns with previous research suggesting that individuals in higher income brackets, who are often employed, have greater access to diverse healthcare options, including facility-based services.^27^ Consequently, they can seek and afford medical treatment beyond home-based care. Conversely, those with lower-income levels often face significant financial and logistical challenges that limit their access to both facility-based and home-based care, such as cost, lack of insurance and limited service availability.^28^ Furthermore, a desire for healthcare services that are seen as more formal or sophisticated may result from socio-economic stability linked to employment.^29^ Lower-income households have consistently shown greater vulnerability to infectious disease outbreaks, which further impacts their access to essential health services.^30^ This underscores the need for targeted interventions to improve access to home-based care for economically disadvantaged populations.

A study found that caregivers earning less than 600 dollars per month bear a notably greater care burden compared to those with higher incomes.^31^ This suggests that financial constraints may hinder the ability of lower-income families to utilise home-based care effectively, which could have an impact on their uptake levels.

Additionally, a study found that patients’ willingness to pay for informal treatment was significantly influenced by their employment status, which serves as a proxy for income.^32,33^ This highlights how inequalities can impact the adoption and sustainability of home-based care, as those with lower incomes may struggle more to manage their medical problems at home.^15,16,34^ Conversely, employed people with stable incomes are generally more willing to invest in home-based care options. These studies emphasise the impact of socio-economic status and income on people’s readiness to support and interact with home-based healthcare services, even though they do not specifically address acceptability of home-based COVID-19 care management. This study found that strategies that improved the awareness of home-based care models included the community’s awareness of the available resources. This aligns with previous studies that assessed home management of COVID-19 and found that knowledge and awareness have a key role in influencing people’s opinions on healthcare options.

Our study also reinforces the role of awareness in increasing the acceptance of home-based care. This aligns with existing literature demonstrating that health literacy and community awareness campaigns significantly influence individuals’ perceptions of alternative healthcare models.^35^ The role of risk communication and community engagement in raising awareness of the advantages of home-based care, such as its affordability and convenience, may help to lessen the stigma and anxieties around it.^36,37^ Furthermore, community-based health education programmes have been found to increase confidence in alternative medical practices, especially during infectious disease epidemics.^38^ Nonetheless, caregivers must educate everyone in the community about home-based care by providing much more awareness about patient care, especially through community-based sensitisation. Integration of Community-based volunteers (CBVs) into the home-based care model supported lower-income households,^30^ while for more affluent areas, the use of telemedicine and digital platforms can be specifically designed.^39^

Increasingly home-based care models are being used to manage other infectious disease outbreaks, as well as non-communicable diseases. The use of community-based volunteers is now being recommended for community case management in cholera outbreaks, prior to admission at treatment centres to provide early care for patients.^40^ Additionally, in non-infectious conditions such as cardiovascular diseases and diabetes, investigators found telehealth use weighted prevalence among patients with CVD.^41^ Policy and programmatic implications from this study suggest the need to create targeted community health strategies for different patient populations in the face of infectious disease outbreaks, while enhancing telehealth and remoting patient monitoring at home. There will be a need to develop standardised training for caregivers and CHWs, while addressing misinformation through targeted health communication strategies.

The study was without limitation. It is postulated that the mistrust of community-based volunteers is a misconception against the use of the home case models. Study focuses on predictors of perceived effectiveness among those already receiving home-based care, rather than determinants of initial acceptance. Additionally, the survey was conducted in April 2024, which may have introduced recall bias, particularly among participants who underwent home isolation between 2020 and 2022. To minimise this potential bias, interviewers used structured questionnaires with specific reference periods and prompts to aid participants’ memory. In addition, data were cross-checked, where possible, with DHO records and case management line lists to validate key information provided by respondents.^42^ The study was not designed to address sustainable financing for home-based care models. However, the strengths of this study were the large sample size and geographic spread of patients that allowed for generalisability across the whole population. The study provides lessons learnt and hindrances for integrating home-based care into broader health security frameworks based on population demographics.

## Conclusion

The findings in our study emphasise the importance of targeted interventions to enhance the uptake and effectiveness of home-based care management. Public health initiatives and strategic information dissemination are essential for raising awareness and building trust in home-based care programmes, especially among those who are unfamiliar with the approach. Home-based healthcare models, whether for chronic illnesses or emergency responses during pandemics, play an important role in reducing healthcare system strain. By enabling home isolation and decentralised patient care, the home-based healthcare models largely contribute to more efficient and high-quality healthcare delivery, ultimately strengthening health system resilience.

## References

[1] Ahmat A, Okoroafor SC, Asamani JS, et al. Health workforce strategies during COVID-19 response: Insights from 15 countries in the WHO Africa Region. BMC Health Serv Res [serial online]. 2024 [cited 2025 Oct 15];24:470. Available from: https://bmchealthservres.biomedcentral.com/articles/10.1186/s12913-024-10942-z?utm_source=chatgpt.com 10.1186/s12913-024-10942-z PMC11017512 38622621

[2] Ziemann M, Chen C, Forman R, Sagan A, Pittman P. Global health workforce responses to address the COVID-19 pandemic: What policies and practices to recruit, retain, reskill, and support health workers during the COVID-19 pandemic should inform future workforce development? [homepage on the Internet]. European Observatory on Health Systems and Policies; 2023 [cited 2025 Oct 15]. Available from: https://www.ncbi.nlm.nih.gov/books/NBK594089/37582188

[3] Filip R, Gheorghita PR, Anchidin-Norocel L, Dimian M, Savage WK. Global challenges to public health care systems during the COVID-19 pandemic: A review of pandemic measures and problems. J Pers Med. 2022;12(8): 1295. 10.3390/jpm12081295 36013244 PMC9409667

[4] Browne C, Tie YC. Promoting well-being: A scoping review of strategies implemented during the COVID-19 pandemic to enhance the well-being of the nursing workforce. Int J Nurs Stud Adv. 2024;6:10077. 10.1016/j.ijnsa.2024.100177 PMC11080544 38746802

[5] Kruk ME, Gage AD, Arsenault C, et al. High-quality health systems in the sustainable development goals era: Time for a revolution. Lancet Glob Health. 2018;6(11):e1196–e1252. 10.1016/S2214-109X(18)30386-3 30196093 PMC7734391

[6] World Health Organisation. Advice for the public [homepage on the Internet]. 2021 [cited 2024 Dec 20]. Available from: https://www.afro.who.int/sites/default/files/Covid-19/Techinical%20documents/Guidance%20_Note%20_for%20Implementation%20concept%20for%20HBIC-final_Com_CM_final-EN.pdf

[7] Alishan S, Ali F, Iqbal Z, et al. Home Management of COVID-19 Patients: A Successful Model in Non-severe COVID-19 Patients in the Developing World. Cureus. 2022;14(1):e21605. 10.7759/cureus.21605. 35228963 PMC8870052

[8] UN Office for the Coordination of Humanitarian Affairs. Zambia Situation Report, 14 September 2020 – Zambia [homepage on the Internet]. ReliefWeb; 2020 [cited 2024 Dec 20]. Available from: https://reliefweb.int/report/zambia/zambia-situation-report-14-september-2020

[9] Khalid A, Ali S. COVID-19 and its challenges for the healthcare system in Pakistan. Asian Bioeth Rev. 2020;12(4):551–564. 10.1007/s41649-020-00139-x 32837562 PMC7424236

[10] Zambia’s COVID-19 home-based care relieves health facilities [homepage on the Internet]. WHO | Regional Office for Africa. 2020 [cited 2025 Apr 02]. Available from: https://www.afro.who.int/countries/zambia/news/zambias-covid-19-home-based-care-relieves-health-facilities

[11] Health workforce policy and management in the context of the COVID-19 pandemic response [homepage on the Internet]. 2021 [cited 2025 Oct 15]. Available from: https://www.who.int/publications/i/item/WHO-2019-nCoV-health_workforce-2020.1

[12] Williams G, Buchan J, Zapata T. Health workforce governance during the COVID-19 pandemic: Learning lessons from Europe. Eur J Public Health. 2022;32(suppl 3):ckac129.041. 10.1093/eurpub/ckac129.041

[13] World Health Organization. WHO-supported COVID-19 home-based care relieves health facilities [homepage on the Internet]. Zambia: World Health Organization. 2021 [cited 2025 Apr 02]. Available from: https://www.afro.who.int/countries/zambia/news/zambias-covid-19-home-based-care-relieves-health-facilities

[14] The hospital at home in the USA: Current status and future prospects. npj Digit Med. 2024;7:48. 10.1038/s41746-024-01040-9 38413704 PMC10899639

[15] Wachtler B, Michalski N, Nowossadeck E, et al. Socioeconomic inequalities and COVID-19 – A review of the current international literature. J Health Monit. 2020;5(suppl 7):3–17. 10.25646/7059 PMC8734114 35146298

[16] Kim PC, Tan LF, Kreston J, et al. Socioeconomic factors associated with use of telehealth services in outpatient care settings during the COVID-19. BMC Health Serv Res. 2024;24:446. 10.1186/s12913-024-10797-4 38594743 PMC11005124

[17] Palinkas LA, Horwitz SM, Green CA, Wisdom JP, Duan N, Hoagwood K. Purposeful sampling for qualitative data collection and analysis in mixed method implementation research. Adm Policy Ment Health Ment Health Serv Res. 2015;42(5):533–544. 10.1007/s10488-013-0528-y PMC4012002 24193818

[18] Mulenga LB, Hines JZ, Fwoloshi S, et al. Prevalence of SARS-CoV-2 in six districts in Zambia in July, 2020: A cross-sectional cluster sample survey. Lancet Glob Health. 2021;9(6):e773–e781. 10.1016/S2214-109X(21)00053-X 33711262 PMC8382844

[19] Katowa B, Kalonda A, Mubemba B, et al. Genomic surveillance of SARS-CoV-2 in the southern province of Zambia: Detection and characterization of alpha, beta, delta, and omicron variants of concern. Viruses. 2022;14(9): 1865. 10.3390/v14091865 36146671 PMC9504048

[20] Impouma B, Mboussou F, Wolfe CM, et al. COVID-19 in the WHO African region: Using risk assessment to inform decisions on public health and social measures. Epidemiol Infect. 2021;149:e259. 10.1017/S0950268821001126 33966683 PMC8712964

[21] Charan J, Biswas T. How to calculate sample size for different study designs in medical research?. Indian J Psychol. 2013;35(2):121–126. 10.4103/0253-7176.116232 PMC3775042 24049221

[22] Cochran WG. Sampling techniques [homepage on the Internet]. 3rd ed. New York: John Wiley & Sons; 1977 [cited 2025 Oct 23]. Available from: https://www.scirp.org/reference/ReferencesPapers?ReferenceID=1390266

[23] Hulley SB, Cummings SR, Browner WS, editors. Designing clinical research. 4th ed. Philadelphia, PA: Wolters Kluwer; 2013, p. 1.

[24] Lachenbruch PA, Lwanga SK, Lemeshow S. Sample size determination in health studies: A practical manual. J Am Stat Assoc. 1991;86(416): 1149. 10.2307/2290547

[25] Kumar R. Research methodology 5th edition [homepage on the Internet]. 2019 [cited 2025 Oct 22]. Available from: https://www.vitalsource.com/za/products/research-methodology-ranjit-kumar-v9781526457103

[26] Council for International Organizations of Medical Sciences (CIOMS). International ethical guidelines for health-related research involving humans [homepage on the Internet]. Council for International Organizations of Medical Sciences (CIOMS); 2016 [cited 2025 Apr 14]. Available from: https://cioms.ch/publications/product/international-ethical-guidelines-for-health-related-research-involving-humans/40523065

[27] NIOSH study examines relationship between employment status, healthcare access, and health outcomes [homepage on the Internet]. 2022 [cited 2024 Dec 07]. Available from: https://www.cdc.gov/niosh/updates/upd-11-18-21.html

[28] CDC. Centers for disease control and prevention. Health care access [homepage on the Internet]. CDC; 2023 [cited 2024 Dec 07]. Available from: https://www.cdc.gov/dhdsp/health_equity/health-care-access.htm

[29] McIntyre D, Thiede M, Birch S. Access as a policy-relevant concept in low- and middle-income countries. Health Econ Policy Law. 2009;4(Pt 2):179–193. 10.1017/S1744133109004836 19187569

[30] Kakietek J, Dayton Eberwein J, Kerr A, Stacey N. To what extent did households in developing countries forgo needed healthcare during the COVID-19 pandemic? Repeated survey estimates from 25 countries in 2020 and 2021. BMJ Public Health. 2024;2(2):e001027. 10.1136/bmjph-2024-001027 40018592 PMC11816704

[31] Janatolmakan M, Naghipour A, Rezaeian S, Khatony A. Predictors of care burden among caregivers of patients with COVID-19. Nurs Open. 2023;10(12):7603–7610. 10.1002/nop2.1999 37743641 PMC10643844

[32] Ramezani-Doroh V, Najafi-Ghobadi S, Karimi F, Rangchian M, Hamidi O. Prediction of COVID-19 patients’ participation in financing informal care using machine learning methods: Willingness to pay and willingness to accept approaches. BMC Health Serv Res. 2024;24:796. 10.1186/s12913-024-11250-2 38987739 PMC11234787

[33] Abel ZDV, Roope LSJ, Duch R, Clarke PM. Access to healthcare services during the COVID-19 pandemic: A cross-sectional analysis of income and user-access across 16 economically diverse countries. BMC Public Health. 2024;24:2678. 10.1186/s12889-024-20147-y 39350210 PMC11443786

[34] Darrat I, Tam S, Boulis M, Williams AM. Socioeconomic disparities in patient use of telehealth during the coronavirus disease 2019 surge. JAMA Otolaryngol Head Neck Surg. 2021;147(3):287–295. 10.1001/jamaoto.2020.5161 33443539 PMC7809608

[35] Nutbeam D. Health literacy as a public health goal: A challenge for contemporary health education and communication strategies into the 21st century. Health Promot Int. 2000;15(3):259–267. 10.1093/heapro/15.3.259

[36] Alishan S, Ali F, Iqbal Z, et al. Home management of COVID-19 patients: A successful model in non-severe COVID-19 patients in the developing world. Cureus. 2022;14(1):1–7. 10.7759/cureus.21605 PMC8870052 35228963

[37] Larki M, Latifnejad RR. Home-based care, the missing link in caring of patients living with HIV/AIDS and their family members: A narrative review. Int J Community Based Nurs Midwifery. 2020;8(3):190–208. 32656272 10.30476/ijcbnm.2020.82771.1085 PMC7334750

[38] Kumar S, Preetha GS. Health promotion: An effective tool for global health. Indian J Community Med. 2012;37(1):5–12. 10.4103/0970-0218.94009 22529532 PMC3326808

[39] Telehealth: A post-COVID-19 reality? [homepage on the Internet]. McKinsey. 2021 [cited 2025 Mar 25]. Available from: https://www.mckinsey.com/industries/healthcare/our-insights/telehealth-a-quarter-trillion-dollar-post-covid-19-reality

[40] Guilford Technical Community, College. Management manual policies. Tackling Cholera Starts in the Community [homepage on the Internet]. Zambia:UNICEF; 2025 [cited 2025 Nov 15]. Available from: https://www.unicef.org/zambia/stories/tackling-cholera-starts-community

[41] Bhatla A, Ding J, Mhaimeed O, et al. Patterns of telehealth visits after the COVID-19 pandemic among individuals with or at risk for cardiovascular disease in the United States. J Am Heart Assoc. 2024;13(17):e036475. 10.1161/JAHA.124.036475 39206726 PMC11646522

[42] Althubaiti A. Information bias in health research: Definition, pitfalls, and adjustment methods. J Multidiscip Healthc. 2016;9:211–217. 10.2147/JMDH.S104807 27217764 PMC4862344

